# Rhythmicity of Intestinal IgA Responses Confers Oscillatory Commensal Microbiota Mutualism*

**DOI:** 10.1126/sciimmunol.abk2541

**Published:** 2022-09-02

**Authors:** Hugo A. Penny, Rita G. Domingues, Maria Z. Krauss, Felipe Melo-Gonzalez, Melissa A.E. Lawson, Suzanna Dickson, James Parkinson, Madeleine Hurry, Catherine Purse, Emna Jegham, Cristina Godinho-Silva, Miguel Rendas, Henrique Veiga-Fernandes, David A. Bechtold, Richard K. Grencis, Kai-Michael Toellner, Ari Waisman, Jonathan R. Swann, Julie E. Gibbs, Matthew R. Hepworth

**Affiliations:** 1 Lydia Becker Institute of Immunology and Inflammation, University of Manchester, M13 9PL, Manchester, United Kingdom; 2 School of Biological Sciences, Faculty of Biology, Medicine and Health, Manchester Academic Health Science Centre, University of Manchester, M13 9PL, Manchester, United Kingdom; 3 Champalimaud Research, Champalimaud Centre for the Unknown, Lisbon, 1400-038, Portugal; 4 Centre for Biological Timing, Faculty of Biology, Medicine and Health, University of Manchester, M13 9PL, Manchester, United Kingdom; 5 Wellcome Centre for Cell Matrix Research, University of Manchester, M13 9PL, Manchester, United Kingdom; 6 Institute of Immunology and Immunotherapy, College of Medical & Dental Sciences, Medical School, University of Birmingham, Birmingham, B15 2TT, UK; 7 Institute for Molecular Medicine, University Medical Center of the Johannes Gutenberg-University Mainz, Mainz, Germany; 8 School of Human Development and Health, Faculty of Medicine, University of Southampton, SO16 6YD, Southampton, United Kingdom

## Abstract

Interactions between the mammalian host and commensal microbiota are enforced through a range of immune responses that confer metabolic benefits and promote tissue health and homeostasis. Immunoglobulin A (IgA) responses directly determine the composition of commensal species that colonize the intestinal tract but require significant metabolic resources to fuel antibody production by tissue-resident plasma cells. Here we demonstrate IgA responses are subject to diurnal regulation over the course of a circadian day. Specifically, the magnitude of IgA secretion, as well as the transcriptome of tissue-resident IgA^+^ plasma cells, were found to exhibit rhythmicity. Oscillatory IgA responses were found to be entrained by time of feeding, and in-part coordinated by the plasma cell-intrinsic circadian clock. Moreover, reciprocal interactions between the host and microbiota dictated oscillatory dynamics amongst the commensal microbial community and its associated transcriptional and metabolic activity, in an IgA-dependent manner. Together our findings suggest circadian networks comprising intestinal IgA, the diet and the microbiota converge to align circadian biology in the intestinal tract and to ensure host-microbial mutualism.

## Introduction

Multiple mammalian species have evolved to maintain a finely balanced relationship with tissue-resident commensal bacteria that is mutually beneficial and critical for tissue homeostasis and the health of the organism. The commensal microbiota confers a multitude of mutualistic functions to mammalian hosts via the provision of complementary metabolic activity, regulation of immune cell maturation and function, and colonization resistance that prevents outgrowth of pathogenic microbes (1–4). Healthy interactions between the host and commensal microbes are dynamically regulated and determined via a complex crosstalk between the microbiota, the intestinal immune system and nutritional cues derived from the diet. Conversely, disruption of this network through changes in lifestyle, diet, infection or antibiotic use can precipitate the onset or progression of metabolic and inflammatory diseases (2, 4–6).

Immunoglobulin (Ig)A is a specialized antibody isotype that acts to regulate commensal bacteria community composition, tissue and niche residence and microbial gene expression (7–10). Within mucosal barrier tissues IgA is the dominant antibody isotype and is produced in a dimeric form bound by a J chain linker that facilitates its selective transport across the intact intestinal epithelium and secretion into the intestinal lumen (7–9). IgA is produced by tissue-resident plasma cells (IgA^+^ PC) predominantly found within the small intestine (11), and at higher quantities than any other antibody isotype at homeostasis – with estimates suggesting that several grams of IgA are produced per day in healthy humans (12). Plasma cells are terminally differentiated antibody-secreting lymphocytes of the B cell lineage, that dedicate the vast majority of their cellular capacity to expanded organelle function required to power antibody translation and secretion (13, 14). In line with this, the differentiation of a class-switched B cell to plasma cell is associated with a huge increase in cell-intrinsic cellular metabolism and nutrient transport (14, 15). Moreover, emerging evidence suggests changes in nutrition and diet can potently perturb IgA responses in the intestinal tract, with consequences for the microbiota and whole-body metabolism (9, 16, 17).

Taken together these findings highlight the significant metabolic requirements of maintaining mucosal antibody responses to reinforce homeostatic host-commensal bacteria interactions and mutualism. To minimize the energetic cost of such metabolically demanding biological axis many species have evolved dynamic regulatory mechanisms – including the regulation of physiological processes through circadian rhythms. Circadian rhythmicity acts to align biological processes with diurnal light cycles and feeding activity, thus temporally regulating biological activity during active periods - associated with feeding activity and potential immune challenges - or periods of rest. Mechanistically this is controlled by a hierarchically layered series of circadian clocks – including the light-sensing suprachiasmatic nucleus of the brain – as well as cell-intrinsic clocks present across a broad range of cell types in peripheral organs, which can be entrained by hormonal, neuronal and dietary cues (18–20). At the molecular level this is controlled by a transcriptional feedback loop mediated by a series of core clock genes that counter-regulate their own transcription – thus imprinting rhythmicity, while also modulating a wider signature of genes to alter cell function (19). Indeed, it is now appreciated that many immune cells exhibit cell-intrinsic circadian-mediated control of cell migration and magnitude of effector functions (21). Furthermore, circadian misalignment - through altered dietary composition and feeding times, jet lag or shift work - has been associated with a number of metabolic and inflammatory diseases, suggesting a better understanding of circadian regulation of immunity will have therapeutic implications. However, the role of circadian rhythms in modulating intestinal immune crosstalk with the microbiota have only recently begun to be explored (22), and yet remain incompletely understood.

Recent advances have also demonstrated diurnal oscillatory behavior within the composition and activity of the commensal microbiota itself, in part imprinted through immune pressures (23–27). Intriguingly it has also been proposed that bacteria may possess analogous circadian clock machinery (28), suggesting circadian rhythmicity and oscillatory biology may have evolved across species to bidirectionally regulate microbial mutualism with the mammalian host. Here, we report diurnal rhythmicity of the secretory IgA response and the IgA^+^ PC transcriptome and demonstrate roles for both the cell-intrinsic circadian clock machinery and cell-extrinsic feeding cues in aligning IgA responses. Critically, bidirectional interactions between the host immune system and microbiota act to entrain rhythmicity in IgA and regulate oscillations in the composition and metabolic activity of the commensal microbiota, thus highlighting circadian regulation of the immune system and microbiota as a key determinant of microbial mutualism.

## Results

### Intestinal IgA responses exhibit diurnal rhythmicity

**Figure 1:**
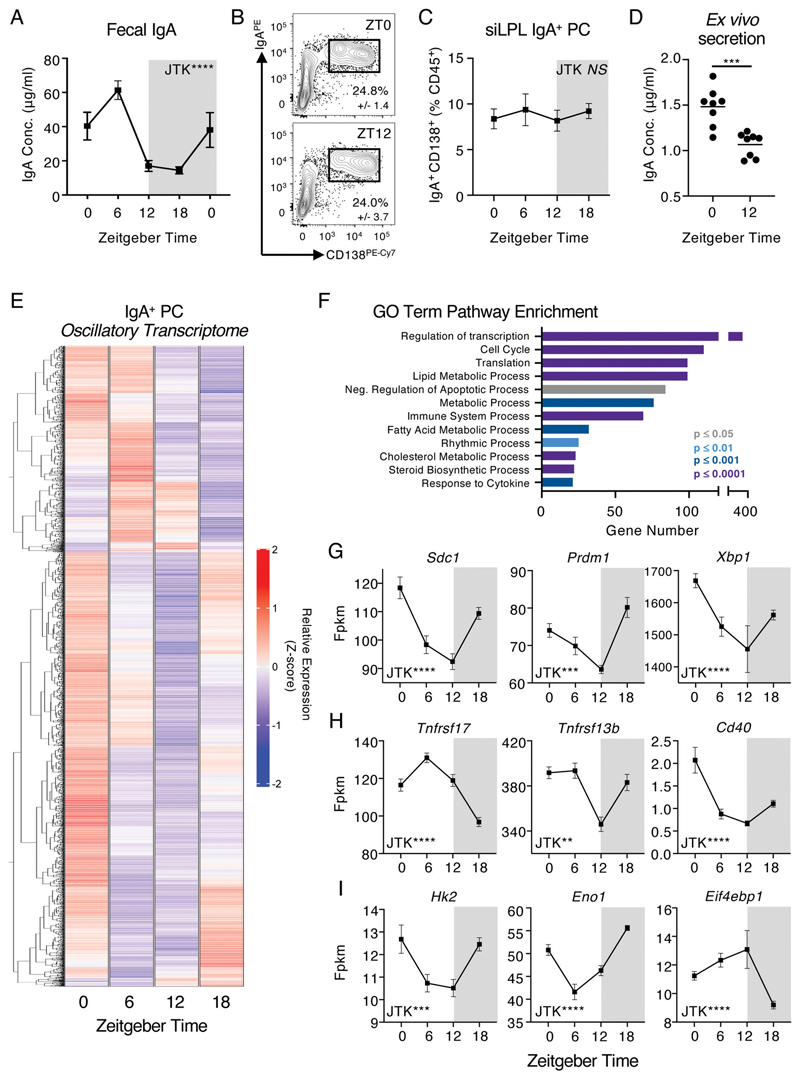
Mucosal antibody secretion and small intestinal IgA^+^ Plasma Cell activity exhibit diurnal rhythmicity. A) Serial fecal sampling of C57BL/6 mice at five 6 hour intervals over a circadian day (ZT 0, 6, 12, 18, 0), *n*=10 (pooled from two independent data sets). Data representative of at least 4 independent experiments. B) Exemplar flow plots of small intestinal CD138^+^ IgA^+^ PC, pregated as Live CD45^+^CD3^-^CD5^-^NK1.1^-^MHCII^+/-^B220^-^IgD^-^, at ZT0 and ZT12 and C) Quantification of IgA^+^ plasma cell frequencies at ZT0, 6, 12 and 18. B+C *n=5* and representative of three independent experiments. D) *Ex vivo* secretion of IgA by sort-purified IgA^+^ PC (from ZT0 and ZT12) cultured for 18 hours. Data pooled from two independent experiments, *n*=8. E) Heatmap of significantly oscillatory genes (JTK Cycle, p<0.01) identified from bulk RNA Sequencing of sort-purified small intestinal IgA^+^ PC taken at ZT0, 6, 12 and 18, z-score of average relative gene expression (fpkm) values of *n*=5 per timepoint. F) GO-Term pathway enrichment analysis on oscillatory gene signatures. Selected relative expression (fpkm) values for oscillatory gene signatures related to G) Plasma Cell function, survival and identitity, H) Extrinsic survival and antibody secretion signals, and I) Cellular Metabolism, values representative of *n*=5 per timepoint. P values were determined using JTK Cycle, with the exception of panel D which was determined via a parametric, unpaired t-test. All data shown as +/- SEM, * p< 0.05, ** p< 0.01, *** p< 0.001, **** p< 0.0001.

We hypothesized that energetically demanding intestinal IgA responses may be subject to diurnal regulation. To test this, we assessed the levels of secretory IgA within the feces of a single cohort of C57BL/6 mice at five time points over a 24-hour day (Zeitgeber times; ZT0, 6, 12, 18, 0). The concentration of IgA detected in the feces was found to exhibit significant and marked variation over the day (Fig. 1A; p<0.0001 by JTK analysis), suggestive of diurnal oscillatory activity, and which remained evident after normalizing for minor variations in total protein content between samples (Fig. S1A). In contrast, we did not observe time of day differences in the frequency or cell numbers of tissue-resident IgA^+^ plasma cells (IgA^+^ PCs) within the small intestinal and colonic lamina propria (Fig. 1B-C and Fig. S1B-D), nor were diurnal oscillations observed in Peyer’s Patch-associated IgA class-switched germinal centre (GC) B cells (Fig. S1E-G). As IgA^+^ PC numbers and intestinal IgA secretion are most enriched in the small intestine (11), we next determined the intrinsic capacity of IgA^+^ PCs sort-purified at different times of the day to secrete IgA *ex vivo*. As expected, IgA^+^ PCs secreted high amounts of IgA into culture supernatants, unlike equal numbers of sort-purified IgD^+^ B cells or IgA^+^ B cells (Fig. S1H). Strikingly, IgA^+^ PCs from ZT0 secreted significantly higher IgA than equal numbers of cells sort-purified at ZT12 (Fig. 1D), suggesting the capacity of IgA^+^ PCs to secrete IgA - as opposed to the numbers of IgA^+^ PCs in the intestine - may determine diurnal rhythms in IgA secretion, as observed in the feces (Fig. 1A).

To further investigate the nature of diurnal regulation of IgA^+^ PC responses, we sort purified small-intestinal IgA^+^ PCs at ZT0, 6, 12 and 18 and performed bulk RNA-seq; of ~16,000 transcripts detected within our samples 2713 genes were found to exhibit highly significant time of day differences and oscillatory patterns after adjusting for false discovery rate (JTK analysis, BHQ <0.01), equivalent to ~16% of the observed transcriptome (Fig. 1E, Fig. S2A - top 50 differentially expressed genes and Data File S1). GO Term enrichment of highly oscillatory genes revealed enrichments in genes involved in Cell Cycle, Protein Translation, Metabolism and Rhythmic Process (Fig. 1F). Notably, oscillations were detected in the expression of key genes involved in IgA^+^ PC phenotype and transcriptional regulation (Fig. 1G), sensing of external activating signals and cell-cell crosstalk pathways known to influence antibody secretory activity (Fig. 1H), and metabolic activity and cholesterol biosynthesis pathways (Fig. 1I, Fig. S2B-G). In line with this we also observed time of day differences in the expression of the PC-associated proteins Xbp1s and CD138 (Fig. S2H-L). Together, these findings suggest that IgA secretion and IgA^+^ PC-intrinsic transcriptional activity within the intestinal tract exhibits diurnal rhythmicity - and provoked the possibility of potential circadian entrainment.

### Cell-intrinsic circadian clock function is required for plasma cell transcriptional rhythmicity, but not rhythmic IgA secretion

**Figure 2:**
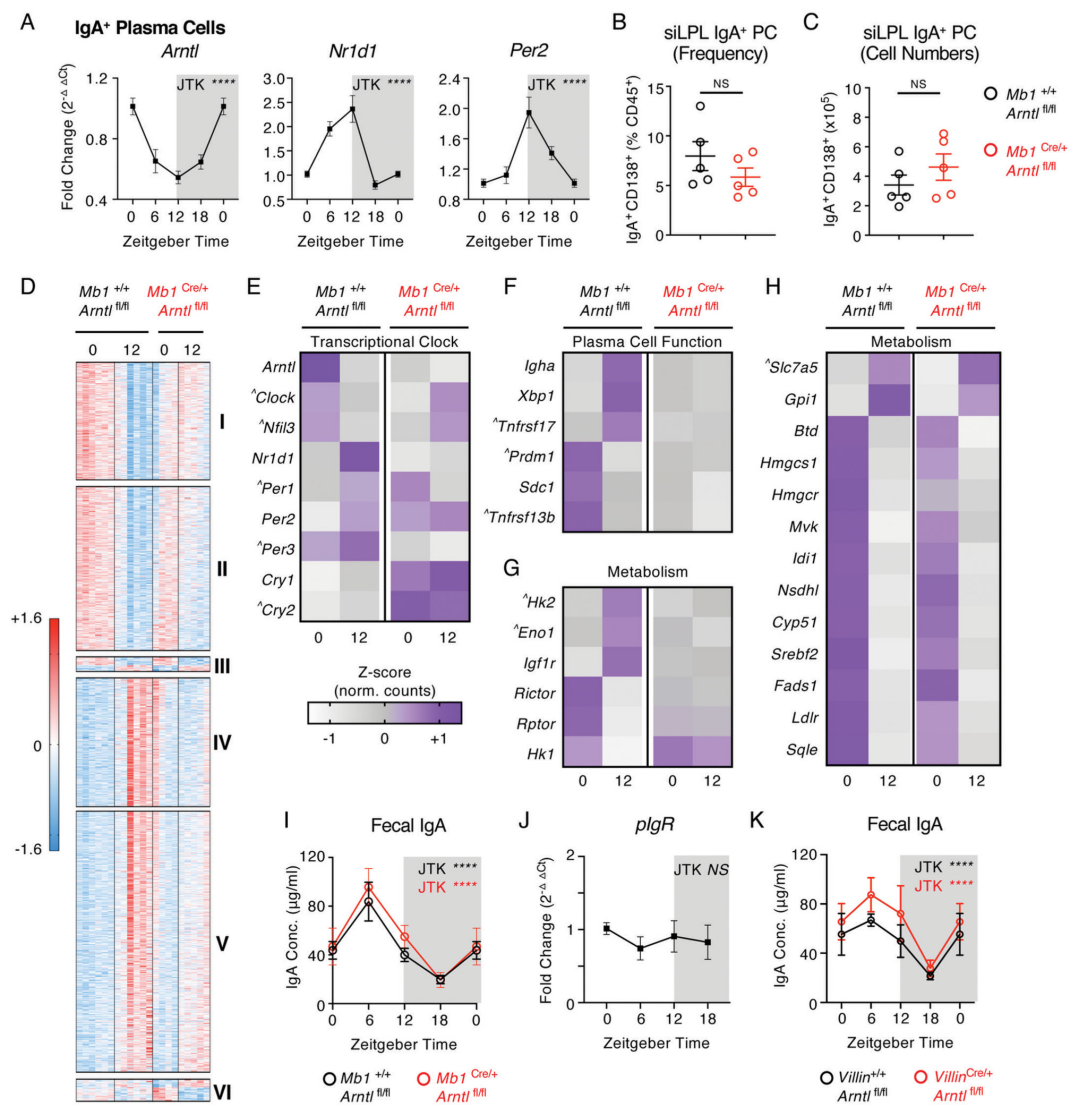
Rhythmic IgA^+^ Plasma Cell activity is in part dictated by the cell-intrinsic circadian clock. A) Relative expression of circadian clock genes in sort-purified small intestinal IgA^+^ PC at ZT 0, 6, 12 and 18 (ZT0 double plotted), determined by RT-PCR. *n*=10 (pooled from two independent experimental cohorts). Data representative of at least 3 independent experiments. B) Frequency and C) numbers of small intestinal IgA^+^ PC in *Mb1*^Cre/+^ x *Arntl*^fl/fl^ mice in comparison to *Mb1*^+/+^ x *Arntl*^fl/fl^ littermate control animals, *n*=5 representative of two independent experiments. D) Heatmap comparison of significantly differentially expressed genes (Benjami-Hochberg adjusted p<0.05) identified by bulk RNA sequencing of sort-purified small intestinal IgA^+^ PC at ZT0 and 12, and found to significantly differ between ZT0 and ZT12 in control animals. Z-scores of relative gene expression (fpkm) values in individual animals of *n*=6 *Mb1*^+/+^ x *Arntl*^fl/fl^ mice and *n*=4-5 *Mb1*^Cre/+^ x *Arntl*^fl/fl^ mice per timepoint. Gene clusters: I+II (decrease in gene expression between ZT0 + ZT12 in controls, loss of suppression in *Mb1*^Cre/+^ x *Arntl*^fl/fl^ mice), III (time of day difference retained in both genotypes), IV+V (increase in gene expression between ZT0 + ZT12 in controls, loss of suppression in *Mb1*^Cre/+^ x *Arntl*^fl/fl^ mice) and VI (enhanced time of day difference in *Mb1*^Cre/+^ x *Arntl*^fl/fl^ mice). E-H) Average z-score values (representative of *n*=6 *Mb1*^+/+^ x *Arntl*^fl/fl^ mice and *n*=4-5 *Mb1*^Cre/+^ x *Arntl*^fl/fl^ mice) in IgA+ PC at ZT0 and ZT12, in respect to E) Circadian clock genes, F) Plasma Cell-associated genes, G+H) Metabolism-associated genes. ^ identifies genes where time of day differences either did not reach statistical significance in control animals in this analysis but were either previously identified in Figure 1 as oscillatory, or are directly related and relevant to the biological pathway described. I) Serial fecal sampling of *Mb1*^Cre/+^ x *Arntl*^fl/fl^ mice and *Mb1*^+/+^ x *Arntl*^fl/fl^ mice at four time points over a circadian day (ZT 0, 6, 12, 18; ZT0 double plotted), *n*=8-9 and pooled from two independent experimental cohorts. J) RT-PCR expression of *pIgR* relative to housekeeping gene in whole small intestinal tissue samples (*n*=5 mice, represenative of two indepednent experiments). K) Serial fecal sampling of *Villin*^Cre/+^ x *Arntl*^fl/fl^ mice and *Villin*^+/+^ x *Arntl*^fl/fl^ mice at four time points over a circadian day (ZT 0, 6, 12, 18; ZT0 double plotted), *n*=5 and representative of two independent experiments. P values for panels A and I-K were determined using JTK Cycle, D-H with Benjami-Hochberg test (DESeq2, see also methods) and panels B+C which with a parametric, unpaired t-test. All data shown as +/- SEM unless otherwise indicated, * p< 0.05, ** p< 0.01, *** p< 0.001, **** p< 0.0001.

Diurnal regulation of oscillatory transcriptional activity and function in both non-immune and immune cells is controlled in part by the cell-intrinsic “clock” - a transcriptional-translational feedback loop mediated by core clock proteins, including CLOCK, Bmal1 (encoded by *Arntl*), Rev-erbα (*Nr1d1*), Period (*Per1/2*) and Cryptochrome (*Cry1/2*). Notably, IgA^+^ PCs were found to have significant oscillations in the expression of *Arntl*, *Nr1d1* and *Per2* by RNA Seq (Fig. S3A), which was independently validated via RT-PCR (Fig. 2A). As expected, expression of *Arntl* within IgA^+^ PCs was found to oscillate in anti-phase to *Nr1d1* and *Per2* over the circadian day, mirroring expression patterns in control liver tissue (Fig. S3B). In contrast, sort-purified naïve IgD^+^ B cells displayed no evidence of rhythmic expression of *Arntl* or *Per2*, although they surprisingly exhibited comparable oscillatory expression of *Nr1d1* (Fig. S3C). To determine the role of this cell-intrinsic circadian clock in regulating IgA secretion within the intestine, we generated conditional knockout mice in which Bmal1 was deleted within the B cell and PC lineages (*Mb1*^Cre^ x *Arntl*^fl/fl^). Efficient deletion of *Arntl* and disruption of associated clock gene transcription was confirmed in IgA^+^ PCs by RT-PCR (Fig. S3D+E), while IgA^+^ PC frequencies and numbers were found to be unaffected by disruption of the cell-intrinsic circadian clock (Fig. 2B+C).

To determine the role of IgA^+^ PC-intrinsic clock gene expression, we performed bulk RNA-seq on sort-purified small intestinal IgA^+^ PCs at ZT0 and ZT12 from *Mb1*^Cre^ x *Arntl*^fl/fl^ and wildtype littermate controls (Fig. 2D), and further confirmed severe disruption of time of day expression of the wider circadian clock gene family following deletion of *Arntl* (Fig. 2E). Analysis of differentially expressed genes revealed significant time of day-dependent signatures in control animals that were either lost (Cluster I and IV), suppressed (Clusters II and V), or retained (Cluster III) in the absence of a functional intrinsic clock (Fig. 2D and Data File S2). Additionally, we observed a time of day gene signature that was significantly enhanced in conditional knockout cells, when compared to controls (Cluster VI). Amongst these signatures we detected a loss of time of day differences in classical IgA^+^ PC-associated genes (Fig. 2F), as also identified in bulk RNA Seq analyses of wild type IgA^+^ PC over four time points (Fig. 1). In contrast, while a proportion of metabolism-associated genes displayed a clear loss of time of day differences in the absence of *Arntl* (Fig. 2G), others – including those involved in glycolysis, amino acid transport, the mevalonate pathway and cholesterol biosynthesis – retained time of day patterns (Fig. 2H), although in some cases the magnitude of this difference was altered or did not reach statistical significance.

Next we determined the impact of disrupted Bmal1-mediated regulation of PC transcription on rhythms in fecal IgA but unexpectedly found oscillations were retained (Fig. 2I). As some time of day signatures in IgA^+^ PC transcription were only partly dependent on intrinsic *Arntl* expression and rhythmicity in IgA secretion was retained, we asked whether IgA secretion into the intestinal lumen could be subject to further circadian regulation at the tissue level. IgA produced by lamina propria-resident PCs requires active transport across the intestinal epithelium by the polymeric Ig receptor (pIgR). However, we failed to detect oscillatory expression of the *Pigr* gene in small intestinal tissue (Fig. 2J), while conditional deletion of *Arntl* in intestinal epithelial cells (*Villin*^Cre^ x *Arntl*^fl/fl^) also failed to perturb rhythmicity in fecal secretory IgA (Fig. 2K). Together these findings suggest that the IgA^+^ PC-intrinsic circadian clock is a major contributor to rhythmic transcriptional activity, but that rhythms in IgA secretion can persist in the absence of intrinsic clock function, indicating additional factors may entrain circadian function.

### Feeding-associated metabolic cues determine the magnitude and rhythmicity of intestinal IgA responses

**Figure 3:**
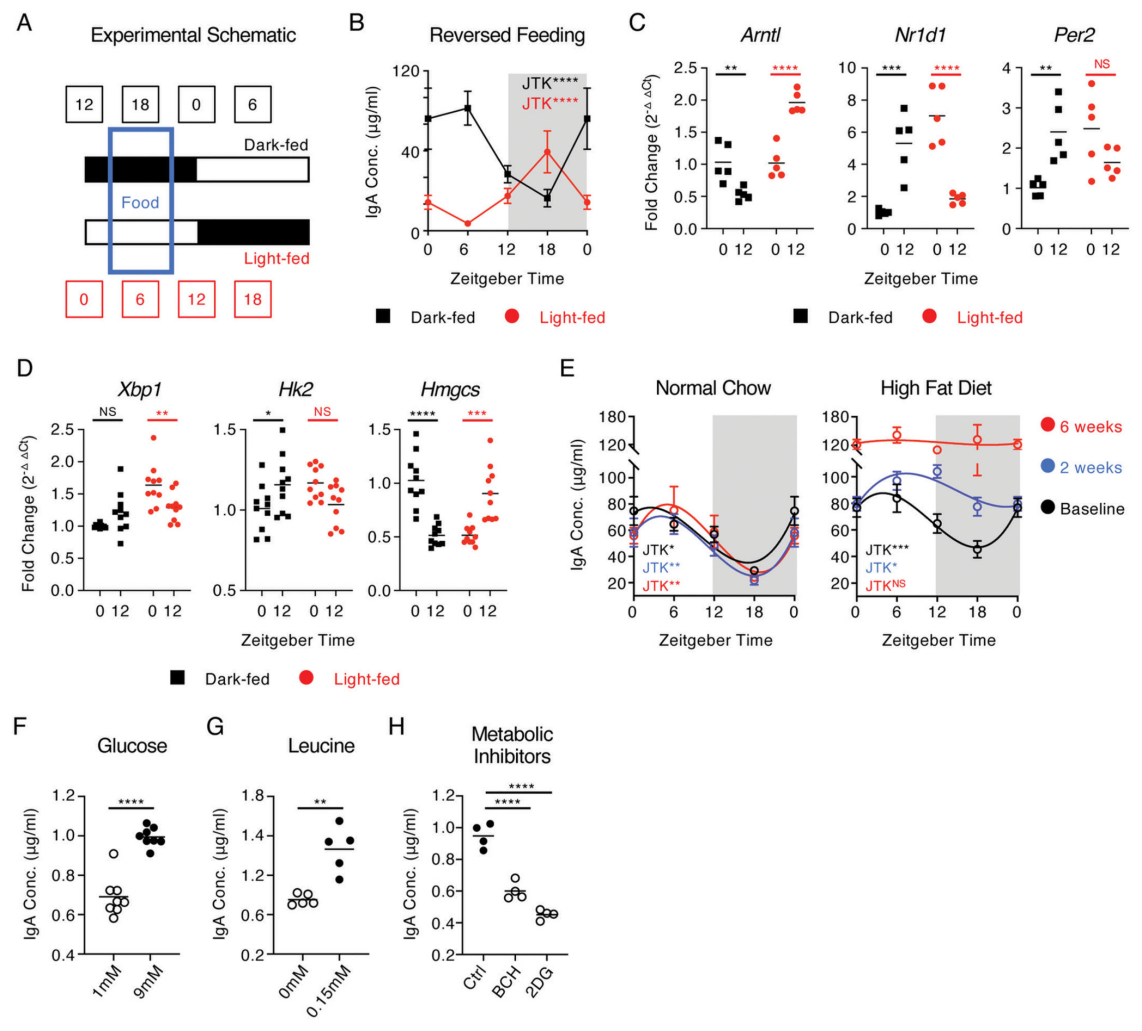
Oscillations in secretory IgA are aligned by feeding cues and cellular metabolic activity. A) Schematic of reversed feeding regimen, B) Serial fecal sampling of light-fed or dark-fed C57BL/6 mice at four 6 hour intervals over a circadian day (ZT 0, 6, 12, 18; ZT0 double plotted), *n*=9-10 (pooled from two independent experimental cohorts). Data representative of at least 4 independent experiments. RT-PCR analysis of C) circadian clock genes (*n*=5 mice per group and representative of two independent experiments) and D) plasma cell associated and metabolic genes (*n*=10 mice per group, pooled from two independent experiments) at ZT0 and ZT12 in sort-purified small intestinal IgA^+^ PC isolated from light-fed or dark-fed mice, *n*=5 per group, data representative of two independent experiments. E) Serial fecal sampling of C57BL/6 mice fed normal chow or high fat diet (HFD at five 6 hour intervals over a circadian day (ZT 0, 6, 12, 18; ZT0 double plotted), taken at baseline, two weeks or six weeks on the indicated diet, *n*=4-5 and data representative of at least 2 independent experiments. F-H) *Ex vivo* secretion of IgA by sort-purified small intestinal IgA^+^ PC cultured with differing concentrations of F) glucose (*n*= 8, representative of pooled data from two independent experiments) G) leucine (*n*= 5, representative of data from two independent experiments) or H) in the presence of metabolic inhibitors (*n*= 4, representative of data from three independent experiments). P values for panels B+E were determined using JTK Cycle. P values for panels C and D were determined using a two-way ANOVA, H with a parametric, unpaired One-Way ANOVA, and F and G with parametric, unpaired t-test. All data shown as +/- SEM unless otherwise indicated, * p< 0.05, ** p< 0.01, *** p< 0.001, **** p< 0.0001.

While cell-intrinsic circadian clocks are important for driving oscillatory immune cell activity, additional exogenous signals can act to entrain these circadian rhythms - most notably feeding cues (22, 29). Moreover, emerging evidence suggests IgA responses are highly sensitive to changes in nutrition and diet (9, 16, 17, 30). To determine whether feeding-associated cues contribute to the entrainment of rhythms in IgA secretion, we utilized light-tight cabinets on reverse 12-hour light:dark schedules in combination with 6 hour periods of feeding restricted to either the dark phase (dark-fed) or light phase (light-fed) (Fig. 3A). Fecal sampling of animals maintained under these conditions at four time points (ZT0, 6, 12 and 18) revealed that dark-fed animals displayed oscillations in fecal IgA similar to that of *ad lib* fed mice (Fig. 3B, Fig. 1A), in line with the largely nocturnal feeding patterns of experimentally housed mice. Strikingly, restriction of food availability to a six-hour window during the light-phase led to a reversal in oscillatory IgA secretion (Fig. 3B and Fig. S4A) – indicating feeding cues act as a key entrainer of IgA secretion in the gastrointestinal tract. Notably, while IgA^+^ PC from dark-fed animals displayed cell-intrinsic time of day differences in clock gene expression comparable with *ad lib* fed mice, reversal of feeding also reversed clock gene expression patterns (Fig. 3C), which was mirrored in the liver (Fig. S4B). Moreover, reversed feeding similarly inverted time of day differences in plasma cell and metabolism-associated genes (Fig. 3D). Conversely, and in line with our findings under *ad lib* conditions (Fig. 1 and Fig. S1), feeding cue-associated regulation of fecal IgA could not be attributed to alterations in IgA^+^ PC or IgA^+^ B cell frequencies in the intestinal tract and associated lymphoid structures (Fig. S4C+D).

Together these findings suggested that feeding-associated cues, such as dietary-derived nutrients and metabolites, may act upstream to entrain cell-intrinsic clock genes - while also acting to regulate cell function through additional mechanisms independent of clock gene expression *per se*. As we observed time of day differences in a series of metabolic genes despite deletion of *Arntl* in IgA^+^ PC (Fig. 2G), we reasoned that feeding cues may further entrain IgA secretion via effects on plasma cell metabolic activity, in concert with clock gene driven regulation of transcription. We thus hypothesized that alterations in dietary nutritional content may perturb rhythms in IgA secretion. As proof of concept we fed mice normal chow or a commercial high fat diet (HFD), to establish a state of overnutrition, and assessed circadian rhythms in IgA secretion at baseline, 2 weeks or 6 weeks later. Animals fed HFD gained a moderate amount of weight over the 6-week period when compared to mice fed normal chow (Fig. S4E), and critically while post-prandial blood glucose was elevated in HFD mice after six weeks (Fig. S4F), no signs of impaired glucose tolerance were observed at this time (Fig. S4G). In contrast, mice fed HFD for a prolonged period of 12 weeks began to exhibit elevated fasting glucose levels (Fig. S4G). Fecal IgA levels consistently exhibited circadian oscillations over a 24-hour period in animals fed normal chow and serially sampled at baseline, 2 weeks and 6 weeks (Fig. 3E). In contrast, while the HFD-fed group exhibited a comparable oscillation in fecal IgA at baseline, the same animals began to exhibit dysregulation of oscillatory IgA secretion following two weeks on HFD, and a complete loss of IgA rhythmicity after 6 weeks (Fig. 3E). Notably however, the overall magnitude of IgA secretion was also increased significantly in mice fed HFD for 6 weeks (Fig. 3E) while, as observed in prior studies (31), feeding of mice on a HFD also led to a blunting of the diurnal food intake (Fig. S4H-I). Taken together these data further suggest HFD may disrupt rhythms in IgA.

Cell-intrinsic metabolic activity and nutrient availability have been demonstrated to be critical determinants of plasma cell survival, function and antibody secretory capacity (14, 15, 32, 33). In line with this concept, we found that small intestinal IgA^+^ PCs exhibited elevated metabolic activity when compared to either IgA^+^ B cells or IgD^+^ B cells derived from the Peyer’s patches (Fig. S5A-J). Notably, IgA^+^ PCs exhibited markedly elevated uptake of the glucose analogue 2NDBG (Fig. S5A-B), expressed higher levels of the solute carrier chaperone protein CD98 – which functionally endowed cells with enhanced amino acid uptake capacity (Fig. S5C-G), and exhibited elevated intracellular lipid content (Fig. S5H-I). The heightened metabolic activity of IgA^+^ PCs was further reflected in extracellular flux assays (Fig. S5J). Nonetheless, the *ex vivo* metabolic activity of IgA^+^ PCs did not significantly differ by time of day (Fig. S5K-N), suggesting that circadian rhythms in IgA^+^ PC function and IgA secretion were not dictated by diurnal changes in the metabolic capacity of plasma cells *per se*. Rather, we hypothesized that changes in nutrient availability - as a result of feeding activity - may act as a rate-limiting factor for antibody secretion by fueling IgA^+^ PC metabolism, and entraining rhythmicity in concert with the cell-intrinsic clock. In line with this hypothesis, the IgA secretory capacity of sort-purified PCs cultured *ex vivo* was found to be sensitive to the nutrient content of culture media, with an increase in glucose from subphysiological (1mM) to physiological (9mM) levels resulting in increased magnitude of IgA secretion (Fig. 3F). Similarly, IgA secretion from cultured PCs was sensitive to the presence of the amino acid leucine in the culture media (Fig. 3G), while pharmacological inhibition of either amino acid transport (BCH) or glycolysis (2DG) conversely reduced the magnitude of IgA secretion (Fig. 3H). Taken together these findings suggest that feeding-associated cues, potentially through changes in nutrient availability, act to entrain and align oscillations in IgA production and the IgA^+^ PC transcriptional circadian clock in-part by fueling cell-intrinsic metabolic activity.

### Reciprocal interactions between host and microbiota determine oscillatory IgA secretion and rhythms in the microbiota to modulate microbial mutualism

**Figure 4:**
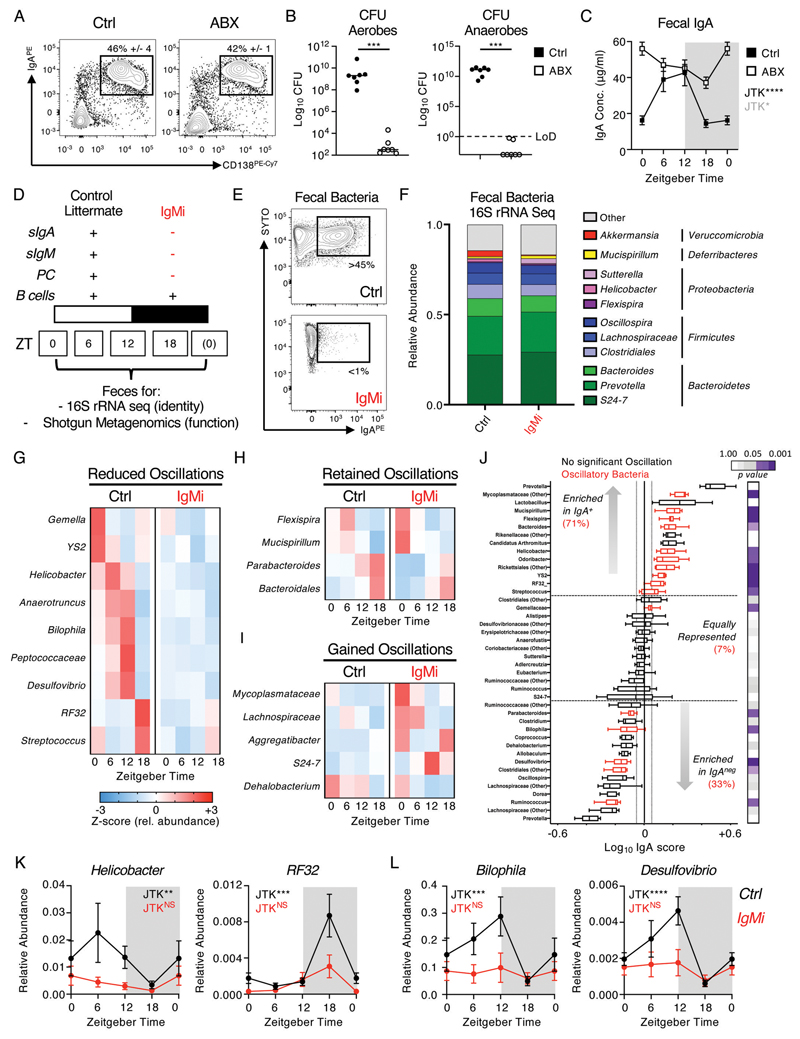
Bidirectional interactons between the microbiota and host IgA responses regulate circadian rhythmicity of commensal microbes. A) Small intestinal IgA^+^ PC frequencies in mice receiving a cocktail of antibiotics (ABX) *ad lib* for 4 days (representative of *n*=5 mice per group and two independent experiments), B) colony forming units (CFU) or commensal microbes measured under aerobic and anaerobic culture conditions (representative of *n*=7 mice per group, pooled from two independent experiments) and C) fecal IgA measured over four circadian time points (ZT0 double plotted) from control and ABX treated mice representative of *n*=5 mice per group and two independent experiments. D) Summary of features of the IgMi mouse model. E) Representative measurement of IgA-binding to fecal bacteria in IgMi mice or littermate wild type control mice (Ctrl). F) Global analysis of average microbiota composition in Ctrl and IgMi animals elucidated by 16S rRNA Sequencing of fecal pellet-derived bacteria. G-I) Z-score heatmaps indicating average relative abundance of significantly oscillatory microbial genera in Ctrl mice and IgMi mice from serially sampled fecal bacteria taken at ZT0, 6, 12 and 18 (JTK cycle p<0.05). J) IgA-Seq analysis of fecal bacteria isolated from Ctrl animals. Bacteria determined to exhibit oscillatory patterns in G-I are highlighted in red and the relative percent enrichment of oscillatory bacteria in IgA^+^ or negative fractions are indicated, legend indicates JTK cycle *p* values. IgA enrichment indicate as log10 score. K+L) Individual data sets for selected bacteria identified as oscillatory in Ctrl animals and perturbed in IgMi mice (ZT0 data double plotted). All 16S rRNA sequencing and IgA Seq data representative of two independent experiments with *n*=4-5 animals per genotype, per ZT time point. P values for panel B were determined using a non-parametric, unpaired Mann-Whitney t-test and panels C and G-L using JTK Cycle. All data shown as +/- SEM unless otherwise indicated, * p< 0.05, ** p< 0.01, *** p< 0.001, **** p< 0.0001.

IgA is a canonical immune regulator of host-commensal microbe interactions and mutualism, and while a significant proportion of the microbiota is bound by secretory IgA the precise impact of IgA on the composition and mutualistic functions of the microbiota has remained incompletely understood. Conversely, emerging evidence suggests that the composition of the microbiota exhibits circadian rhythmicity which is in part dictated by host immune circuits, but is also likely to provide cues that align rhythms in immune responses due to the inherently linked nature of mucosal immune responses (22–25, 27, 34). To first address the role of the microbiota in dictating IgA rhythmicity we investigated IgA responses in Germ Free animals, however as previously reported the absence of a microbiota dramatically reduces the generation of IgA^+^ PC in the intestinal lamina propria (Fig. S6A-B). In line with this, IgA levels were markedly reduced in the feces compared to SPF controls (Fig. S6C). To further clarify the role of microbiota in regulating the rhythmicity of the IgA response, we transiently treated SPF mice with a cocktail of antibiotics which successfully depleted commensal microbes without significantly altering the intestinal IgA^+^ PC pool (Fig. 4A-B). Strikingly, antibiotic treatment led to a clear disruption of normal rhythmicity in fecal IgA, although perturbed IgA production remained statistically significant by JTK cycle analysis - suggesting IgA rhythms were dramatically altered but not absent *per se* (Fig. 4C).

While these observations suggest the microbiota itself is required for homeostatic IgA rhythmicity, we proposed that conversely IgA responses against the microbiota could in turn regulate oscillations in microbial composition. The dissection of the precise roles of IgA in regulating the commensal microbiota have been hindered by the observed generation of compensatory IgM responses in both *Igha*-knockout mice and IgA-deficient humans, which bind to a comparable repertoire of commensal bacteria (11, 35–37). To circumvent this issue and determine whether circadian oscillations in intestinal IgA impact upon the commensal microbiota we utilized IgMi mice, which lack the ability to class switch and secrete antibody yet retain a mature B cell compartment (38–40)(Fig. 4D). Thus, this model allowed us to study the microbiota in the absence of both secretory IgA and any other mucosal antibody isotypes transported into the intestinal lumen in the absence of IgA that may fully or partially compensate. As expected IgMi mice lacked detectable fecal IgA by ELISA when compared to littermate control animals (Ctrl) (Fig. S6D), and furthermore lacked IgA-bound bacteria as determined by flow cytometry (Fig. 4E, Fig. S6E). Next, we serially collected fecal samples from IgMi and littermate control animals over multiple circadian time points and performed 16S rRNA sequencing. We confirmed time of day differences in the total abundance of commensal bacteria as has been previously reported (23–25), but found oscillations in total microbial abundance to be unaffected in IgMi mice (Fig. S6F). Similarly, and in line with previous findings (39), we did not observe any dramatic changes in the global composition of fecal bacteria at the phylum or genus level when analyzing the global composition of the microbiota across the mean of all samples by genotype (Fig. 4F; data shows average of combined timepoints). One notable exception was a clear reduction in the abundance of *Akkermansia* in IgMi mice (Fig. 4F and Fig. S6G), suggesting IgA binding may favor colonization of this mucosal-dwelling microbe. However, analysis by *Zeitgeber time* identified rhythms within the commensal microbiota, in line with previous reports (23–25, 27, 34). Consistent with these prior studies we were able to identify circadian rhythmicity in a number of bacteria genera including *Mucispirillum, Helicobacter*, *Peptococcaceae*, *Desulfovibrio* and *Bilophila* (Fig. 4G-I, K-L), while other major bacterial genera demonstrated no observable time of day differences (Fig. S6H). Critically, we identified a signature of rhythmic bacteria that lost circadian rhythmicity in the absence of IgA (Fig. 4G, K-L, Fig. S6J), although others retained or gained rhythmicity in IgMi mice (Fig. 4H-I). To determine whether the loss of bacterial rhythmicity in IgMi mice correlated with direct IgA-binding we first quantified IgA binding of total fecal commensal microbes by flow cytometry, but observed no significant differences in the total proportion of the microbiota labelled by IgA over time (Fig. S6M-N). Despite this, by sequencing of IgA-bound versus unbound bacteria (IgA-Seq) we identified an enrichment (71%) of oscillatory microbes (*red*) amongst bacteria identified to be preferentially IgA bound in control animals (Fig. 4J). Notably, many of IgA-bound bacteria also demonstrated a loss of rhythmicity (Fig. 4G-K), or changes in circadian phase (Fig. S6K) in the absence of mucosal antibody. Surprisingly, while we also observed a small subset of oscillatory bacteria that were preferentially enriched in the IgA negative fraction and unperturbed in IgMi mice (Fig. S6L), we also detected some bacteria that were not directly bound by IgA yet lost rhythmicity in IgMi mice (Fig. 4L) – suggesting circadian regulation of commensal microbiota constituents is likely highly complex and potentially subject to reciprocal interactions and competition for niches that alter the relative abundances of microbial species within the community. Thus, we were able to identify oscillations in the abundance of a number of commensal bacteria that were dependent upon IgA, the secretion of which is itself regulated in a circadian manner.

**Figure 5:**
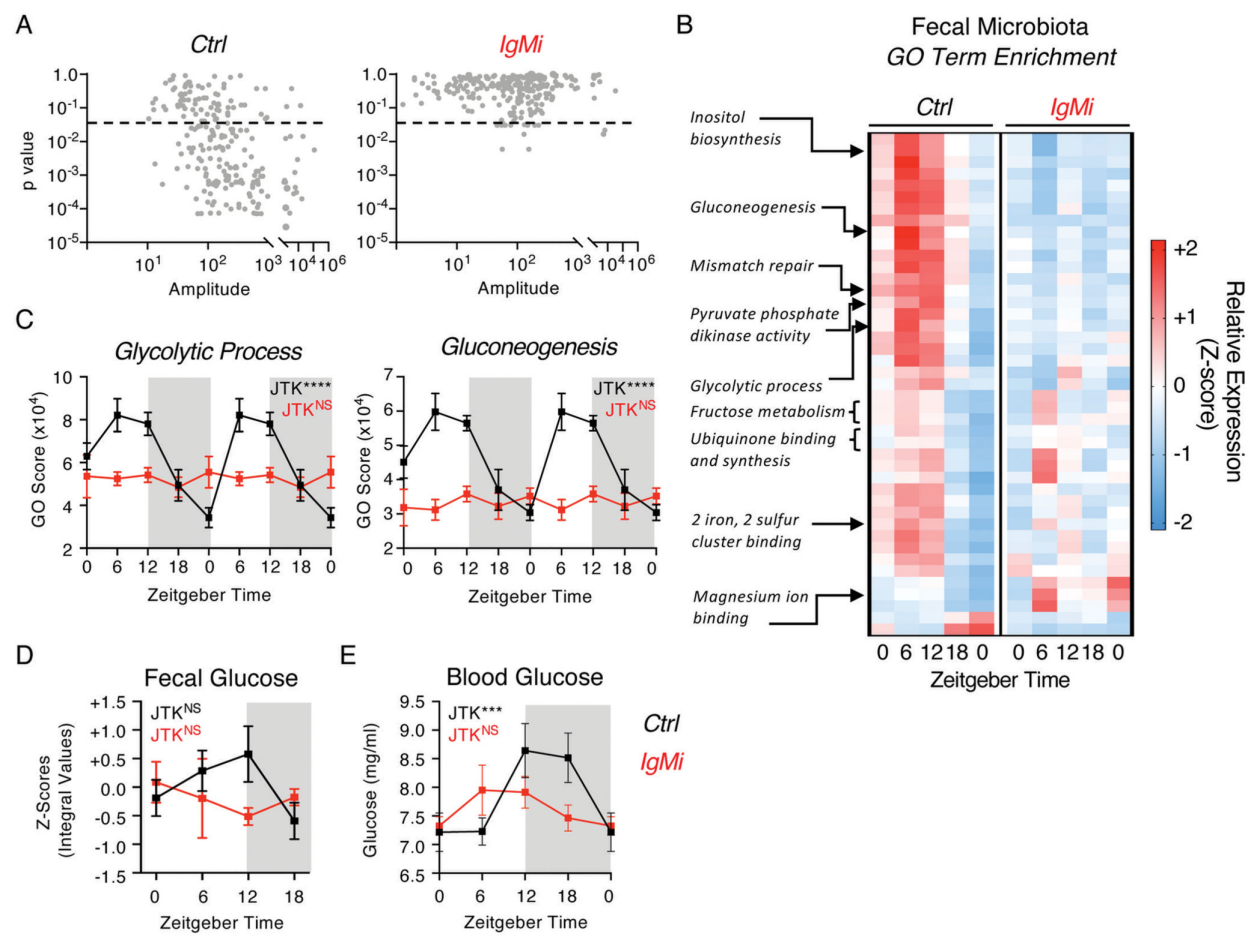
Mucosal antibody regulation of microbiome circadian rhythmicity modulates nutrient and metabolite availability and uptake. A) JTK analysis of GO Term pathway scores identified by shotgun metagenomics of serially sampled feces of Ctrl and IgMi mice over five 6 hour intervals over a circadian day (ZT 0, 6, 12, 18, 0), *n*=5 per group per timepoint, and representative of a single experiment. Significance cutoff = p<0.05. B) Z-score heatmap of average GO Term scores identified to be significantly oscillatory in Ctrl mice and perturbed in IgMi mice by JTK cycle analysis (p<0.05), and C) select exemplar pathways double plotted. D) Glucose levels in serially sampled feces of Ctrl and IgMi mice over five 6 hour intervals over a circadian day (ZT 0, 6, 12, 18, 0), *n*=5 per group per timepoint, and representative of a single experiment. E) Glucose levels in serially sampled blood of Ctrl and IgMi mice over five 6 hour intervals over a circadian day (ZT 0, 6, 12, 18; ZT0 double plotted), *n*=8-12 per group per timepoint, representative of data pooled from three independent experiments. P values determined via JTK Cycle. All data shown as +/- SEM unless otherwise indicated, * p< 0.05, ** p< 0.01, *** p< 0.001, **** p< 0.0001.

While this provides evidence for a circadian role for IgA in regulating the composition of commensal bacteria, the consequences of this for the mutualistic functions of the microbiota and the mammalian host were unclear. Thus, we further performed shotgun metagenomics on serially sampled fecal bacteria from littermate control mice over five distinct time points (ZT0, ZT6, ZT12, ZT18 and a second ZT0, within the same 24-hour period). Analysis of functional GO-Terms in wild type control littermates predicted that a significant proportion of predicted bacterial functional pathways undergo circadian oscillation (Fig. 5A). Strikingly, IgMi mice exhibited a near-complete loss of highly oscillatory GO-Terms when compared to littermate controls (Fig. 5A-B and Data File S3). Many of the microbial GO-Terms that were found to be oscillatory in control animals and lost in IgMi mice related to metabolic processes, including *Glycolytic Process* and *Gluconeogenesis* (Fig. 5B+C, Fig. S7A-B), suggesting the presence of IgA may promote rhythmicity in microbial metabolism and liberation of nutrients from the diet. We also identified a small number of GO-Terms that indicated alterations in basic microbial biology, including several that in contrast were predicted to gain oscillations in the absence of IgA, including bacterial *Flagellum Assembly* and *Extrachromosomal Circular DNA* (Fig. S7C). Next, in order to determine whether changes in microbial function and metabolic activity altered nutrient availability within the intestine we performed metabolomics on fecal samples from IgMi mice and littermate controls. We observed evidence of time of day differences in the relative abundance of glucose in the feces over the course of a day, which were blunted in the absence of mucosal antibody (Fig. 5D), and to a lesser extent in short chain fatty acid availability (Fig. S8A), while availability of succinate exhibited comparable time of day differences regardless of the presence or absence of mucosal antibody (Fig. S8A). Despite changes in fecal metabolite levels, we confirmed that IgMi mice retained comparable circadian patterns in food intake (Fig. S8B), suggesting differences could not be attributed to changes in feeding behavior. To determine the potential impact of circadian changes in intestinal metabolite availability on the host we placed mice in metabolic cages (CLAMS) but found no evidence for major dysregulation of whole-body metabolism and energy usage (Fig. S8C-D). However, we observed perturbed time of day differences in circulating glucose in the blood of IgMi mice (Fig. 5E), which mirrored predicted microbial metabolic activity and glucose abundance in the feces, thus suggesting that circadian IgA regulation of microbial function may modulate time of day differences in metabolite availability and/or uptake by the host.

## Discussion

The complex interplay between the microbiota, intestinal immune system and diet is increasingly understood to be at the center of a broad range of inflammatory, metabolic and systemic pathologies – and an increasing driver of morbidity and mortality in the industrialized world. Here we provide evidence of circadian regulation of a major mucosal immune pathway with critical functions in regulating host-microbiota crosstalk, which we hypothesize may have provided evolutionary benefit by balancing the energetic cost of an intestinal immune response with optimal orchestration of microbial mutualism. Specifically, we report diurnal secretion of IgA - in line with previous observations (41, 42) - and demonstrate feeding as a major cue in aligning rhythms in mucosal antibody secretion. Furthermore, we demonstrate that the absence of IgA significantly impacts upon oscillations in the relative composition and activity of intestinal microbes. Together, our findings suggest a combination of cell-intrinsic circadian clocks and cell-extrinsic, feeding cues entrain rhythms in IgA, and in turn modulates the commensal microbiota (Fig. S9).

Here we observed the presence of an active transcriptional circadian clock in IgA^+^ PC, which was surprisingly largely absent in naïve B cells indicating circadian clock rhythmicity may be imprinted during B cell activation or class switching. Nonetheless, we did observe *Nr1d1* expression in naïve B cells which may suggest roles for Rev-erb alpha beyond circadian regulation, as described for other immune cells (43, 44). However, while we observed IgA^+^ PC-intrinsic oscillations in canonical clock genes, and significant disruption of clock gene expression upon deletion of *Arntl*, rhythmicity in luminal secretory IgA was retained - suggesting Bmal1 is dispensable for oscillations in IgA secretion and indicating the potential for additional peripheral cues. In line with an emerging body of evidence (45, 46), we identified feeding events as a critical cue to entrain IgA responses and PC rhythmicity. Intriguingly reversed feeding experiments also similarly perturbed time of day differences in circadian clock genes and other highly rhythmic genes, suggesting feeding cues may link extrinsic and intrinsic circadian timing in IgA^+^ PC. Nonetheless, we cannot rule out roles for circadian clock gene regulation independent of Bmal1. For example, other clock components have been reported to retain rhythmicity in the absence of Bmal1, while Rev-erbα has been attributed clock-independent roles as a transcription factor (43). Moreover, *Xbp1* – which was also found to be rhythmic in IgA^+^ PC and entrained by feeding here – has recently been described to induce oscillatory gene expression independent of core clock genes (47).

Our findings provide further evidence for feeding as a critical entraining cue for peripheral circadian rhythms in intestinal immune cells. Elevations in IgA in fecal pellets aligned with periods of feeding in both *ad lib* and reverse-fed mice, although of note we observed a lag effect whereby elevations in IgA also persisted into the relative fasting phase, most likely due to the time delay between food ingestion, liberation and absorption of nutrients, changes in IgA^+^ PC function, and subsequently detection of secreted IgA following transit of fecal pellets along the intestinal tract. Indeed, both long-term undernutrition or chronic overnutrition can alter the generation of IgA responses in the intestinal tract, suggesting a complex interplay between nutrition, circadian rhythms and mucosal antibody responses and host-commensal mutualism (9, 16, 17, 22, 23, 25, 27, 29, 30). Despite these advances the precise molecular mechanism through which feeding modulates IgA secretion in the gut remains unclear. One hypothesis is that elevated nutrient availability following feeding acts a key determinant of the ability of intestinal plasma cells to fuel enhanced antibody secretion, and in line with this we demonstrated altering nutrient or metabolite levels was sufficient to change the magnitude of IgA secretion *ex vivo*. Additionally the feeding of a high fat diet (HFD) disrupted rhythms in IgA, although it should be noted that HFD has marked effects on circadian feeding activity (31), the microbiota and intestinal inflammation – in addition to altering nutritional content – all of which could feasibly contribute to this phenotype given the interconnected nature of diet, microbiota and intestinal immunity. Nutritional cues can be sensed and propagated via a number of intracellular metabolic signaling pathways and transcriptional regulators, but also by neuronal and hormonal circuits (48). Thus, further studies are required to determine the precise intracellular sensing pathways that connect feeding events with rhythmic IgA+ plasma cell circadian clock-driven transcription and secretory function. Nonetheless, IgA responses and plasma cells are known to be particularly sensitive to changes in nutrition (9, 16, 17), and studies to decipher the mechanistic basis through which diet alters intestinal IgA production may provide critical insights into the etiology of diet driven changes in the microbiota that predispose to inflammatory and metabolic disease.

In line with several previous studies a lack of IgA did not result in a marked global dysbiosis *per se*, although we observed a loss of *Akkermansia* spp. in line with current understanding that in many cases IgA promotes host mutualism with mucosal-dwelling commensals (49–51). Strikingly however, we were able to recapitulate seminal observations made by other groups who reported diurnal oscillations in many of the same commensal microbes – including *Mucispirillum*, *Peptococcaceae* and *Streptococcaceae* spp (23–25, 27, 34). Critically, as in previous studies, these oscillations in bacterial constituents further manifested as time of day regulation of commensal function and broader microbial biology – most notably in pathways orchestrating nutrient metabolism, bacterial replication and pathogenicity (23, 25, 27). The role of IgA in impacting upon microbial transcription and functional biology – as opposed to composition - has remained relatively poorly understood, although recent advances have begun to delineate the role of IgA binding in regulating microbial metabolic activity, motility and fitness (10). Here, we demonstrate that lack of IgA secretion causes a loss in diurnal oscillations at the level of both composition and microbial activity predicted via metagenomic sequencing, and build upon previous findings in the field to suggest IgA binding has key roles in modulating bacterial gene expression. For example, we found that lack of IgA led to a gain in flagellum assembly over the circadian day, supporting findings that IgA binding can suppress bacterial flagellum expression and thus, motility (52). One notable observation was the circadian regulation of microbial pathways of glucose metabolism and glucose availability both within the intestine and circulation – adding to previous findings that IgA may be an important immune pathway in the regulation of glucose metabolism and risk of metabolic disease (16). Despite these advances the precise mechanism through which oscillations in IgA alter rhythmic microbial composition and function remain to be elucidated but suggest host-microbiota interactions are highly dynamic over the course of a circadian day. Nonetheless, our findings provoke the hypothesis that circadian immune regulation of the microbiota may act to promote mutualism, metabolite availability and metabolic health, which together with recent advances (53) suggest IgA acts to determine host exposure to microbially-derived metabolites.

Our observations complement and expand upon other recent studies that together suggest circadian regulation may be a common feature of tissue-resident intestinal immune cells that constitutively act to maintain healthy interactions with commensal bacteria (26, 27, 43, 44, 54–57), and that immune pressure may partially imprint rhythmicity on the microbiota itself to confer mutualistic benefits for the host over the daily light:dark cycle, including ensuring energetic and metabolic efficiency aligned with feeding activity. An increasing body of evidence has begun to link lifestyles that disrupt circadian rhythmicity and microbial rhythms with the onset and progression of human inflammatory and metabolic diseases, including type 2 diabetes (58), and thus an increased understanding of circadian immune regulation will be critical to harness the full potential of the emerging field of circadian medicine (59, 60)

## Materials and Methods

### Study Design

The study was designed to determine whether the magnitude, phenotype or transcriptome of the IgA Plasma Cell response in the small intestine was subject to diurnal variation. In addition, we aimed to uncover the intrinsic and extrinsic cues that regulate the magnitude of IgA responses over the course of a 24-hour period and to determine the requirement for IgA in contributing to circadian changes in the composition, dynamics and predicted activity of the intestinal commensal microbiota. All *in vivo* experiments were performed a minimum of two times with animal numbers per group indicated in the respective figure legends. All transgenic mouse lines were age- and sex-matched and co-housed with control littermates within each individual experiment. Mice of different genotypes were randomly assigned to cage and group and studies were unblinded.

### Mice

Age and sex-matched C57BL/6 mice were purchased from Envigo laboratories. *Mb1*^Cre^ x *Arntl*^fl/fl^ were originally crossed by Kai-Michael Toellner (University of Birmingham) and are available via Jackson laboratories (*Mb1*^Cre^ - strain 020505; *Arntl*^fl^ – strain 007668). *Villin*^Cre^ x *Arntl*^fl/fl^ were maintained within the Centre for Biological Timing at the University of Manchester, Villin^Cre^ are available via Jackson laboratories (strain 004586). IgMi mice were a kind gift from Ari Waisman (IMB Mainz) and are available subject to collaborative agreement. All transgenic mouse experiments were performed using cohoused littermates, of mixed genotype, and under specific pathogen free conditions with *ad libitum* feeding as 12h:12h light:dark cycle at the University of Manchester, United Kingdom, unless otherwise specified. In some cases, mice received irradiated High Fat Diet (Research Diets; D12492i; 60% Kcal from fat) *ad lib* for up to 12 weeks. Where indicated experimental cages were placed in controlled light-tight cabinets under opposing 12-hour light:dark cycles to facilitate investigation of circadian rhythms. In some experiments mice were placed in bespoke housing for the measurement of metabolic readouts and feeding as detailed below. Germ Free mice were bred and maintained in the University of Manchester Axenic and Gnotobiotic Facility. All other animal experiments were performed under Specific Pathogen Free (SPF) in single ventilated cages conditions and under license of the U.K. Home Office and under approved protocols at the University of Manchester.

### Tissue Processing

Small intestinal lamina propria lymphocyte preparations were prepared by opening longitudinally and removing the Peyer’s patches, associated fat and luminal content by gently shaking in cold PBS. Epithelial cells and intra-epithelial lymphocytes were removed by shaking tissues in stripping buffer (1 mM EDTA, 1 mM DTT and 5% FCS) for two rounds of 20 min at 37°C. Lamina propria lymphocytes were isolated by digesting the remaining tissue in 1 mg/mL collagenase D (Roche) and 20 µg/mL DNase I (Sigma-Aldrich) for 45 min at 37°C. Liberated cells were then extracted by passing the tissue and supernatant over a 70μm nylon filter and centrifuged to isolate lamina propria lymphocytes. Isolated Peyer’s patches were processed by passing them through a 70μm nylon filter. In a small number of cases Peyer’s patches were retained during intestinal tissue digest to facilitate concurrent analysis of tissue-resident plasma cells and B cell subsets.

### Flow Cytometry

Single cell preparations were stained with antibodies against surface or intracellular markers as indicated in Supplementary Table 1. Specific conjugates used are indicated within Figures. Dead cells were excluded from analysis using the LIVE/DEAD Fixable Aqua Dead Cell Stain (Life Technologies). Intracellular staining was performed using the FoxP3 fixation/permeabilization kit (ThermoFisher). Samples were acquired using a BD Fortessa Cytometer, and analysed with FlowJo (TreeStar).

### Bacterial flow cytometry

Feces were collected in Fast Prep lysing Matrix A tubes (MP Biomedicals), resuspended in 1ml of PBS per 100mg fecal material and incubated at 4°C for 20 min. Bacterial suspensions were resuspended in a final volume of 2 ml PBS and incubated at 4°C for 20 min. Samples were homogenized in a FastPrep-24 Tissue homogenizer (MP Biomedicals) for 30s. After homogenization, samples were centrifuged at 50 x *g* for 15 minutes at 4°C to remove debris and the bacteria-containing supernatant transferred through 70μm filters into a new tube. Bacteria were washed in FACs buffer (PBS, 2% FCS, 5mM EDTA) and pelleted at 8000 x *g* for 5 min. For flow cytometry, bacterial pellets were resuspended in 100μl FACs buffer containing SYTO 9 green fluorescent nucleic stain (Life Technologies) (10μM), incubated at 4°C for 15 minutes, and subsequently stained with 1μg/ml of an anti-mouse IgA-PE antibody (clone mA-6E1, eBioscience) for 30 min at 4°C. Samples were thoroughly washed and acquired on a BD Fortessa flow cytometer.

### Cell-sorting and *ex vivo* culture assays

*Ex vivo* culture assays were performed on sort-purified IgA^+^ Plasma Cells, IgA^+^ or IgD^+^ B cells, or other control populations isolated from the small intestines and/or Peyer’s patches of female C57BL/6 mice unless otherwise indicated. Kynurenine uptake was assessed as previously reported (61). Briefly, after surface antibody staining, 2x10^5^ cells were resuspended in 200μl warmed Hanks Balanced Salt Solution (HBSS; Sigma, UK), and 100μl of HBSS, or BCH (40mM, in HBSS), or leucine (20mM, in HBSS), was added to appropriate samples. Kynurenine (800μM, in HBSS) was then added and uptake subsequently stopped after 4 minutes by adding 125μl 4% PFA for 30min at room temperature in the dark. After fixation, cells were washed twice in HBSS and then resuspended in HBSS prior to acquisition on the flow cytometer. For assessment of 2-NBDG uptake *in vitro*, 1x10^6^ small intestinal cells were cultured in glucose-free DMEM medium (Agilent, USA) supplemented with 2mM L-glutamine and 100μM 2-NBDG (Thermo Fischer, USA) for 10 minutes at 37°C. Surface antibody staining of samples was then performed and acquisition of samples on the flow cytometer was undertaken within 2 hours. For assessment of lipid accumulation within cells *in vitro*, 1x10^6^ small intestinal cells were cultured in glucose-free DMEM medium (Agilent, USA) supplemented with 2mM L-glutamine and LipidTOX™ (Thermo Fischer, USA) for 30 minutes at 37°C. Cells were then washed, surface antibody staining of samples was then performed and acquisition of samples on the flow cytometer was undertaken within 2 hours.

### Antibiotics Treatment

Mice were treated for a total of 6 days with an antibiotic cocktail containing 1mg/ml ampicillin, 1mg/ml neomycin, 1mg/ml gentamicin, 0.25mg/ml metronidazole and 0.5mg/ml vancomycin in water supplemented with artificial sweetener tablets for palatability. After 3 days of antibiotics, depletion of the microbiota was confirmed by counting colony forming units (CFU) of homogenized fecal pellet supernatants on Brain Heart Infusion (BHI) medium (CM1134, Thermo Fisher) plates cultured under both aerobic conditions at 37°C for 48 hours, or incubated in Wilkens-Chalgren anaerobe medium enriched with 10% horse blood (CM1135, Thermo Fisher) under anaerobic conditions in an Whitley MG500 anaerobic incubator with 10% (v/v) H2, 10% CO2 and 80% N2 for 37°C for 48 hours. Feces of antibiotic treated mice were subsequently collected over four circadian time points beginning on day 4 to measure IgA.

### ELISA

Mouse fecal IgA titers were measured using the Mouse IgA ELISA Quantitation Set (Bethyl Laboratories) following manufacturers’ instructions. Fecal samples were serially diluted and optimal dilutions and concentration were determined based via a standard curve. For core data sets an additional BCA assay (Pierce Coomassie Plus (Bradford) Assay Kit, Thermo Scientific) was performed on fecal extracts to measure total protein, and IgA concentrations normalized.

### Metabolic inhibitor assays

Sort-purified IgA+ PCs isolated from the small intestinal lamina propria were incubated (10^4^ cells/ well) in either leucine free media (US Biological, USA) or glucose free media (Gibco, UK), with IL-6 (10ng/ml) (Peptrotech, USA) and BAFF (200ng/ml) (Biolegend, UK), supplemented with differing concentrations of leucine, or glucose (both Sigma, UK). To determine the effects of inhibiting nutrient uptake or metabolic signaling on IgA secretion, sort-purified IgA+ PCs isolated from the small intestinal lamina propria were cultured (10^4^ cells/well) as above with or without the addition of metabolic inhibitors including pp242 (500nM), BCH (10mM) and 2-Deoxy-D-glucose (2DG) (1mM) (all Sigma, UK). Cells were incubated for 16 hours at 37°C, following which culture supernatants were removed and IgA concentrations determined by ELISA. Cell viability was determined under different culturing conditions, by either using a hemocytometer or flow cytometry.

### Extracellular Flux Analysis

Extracellular flux analysis (Agilent, USA) was performed with replicates of 150,000 sort-purified IgA+ PCs isolated from small intestinal lamina propria or IgD+ B cells isolated from Peyer’s patches. Cells were adhered to each well of the Seahorse plate (Seahorse/Agilent, USA) using CellTak (Corning, USA). Cells were rested in Seahorse medium (glucose-free DMEM) at 37°C without CO2 for at least 30 minutes prior to the run. For the test, Seahorse XF medium was supplemented with 2mM of L-glutamine (Sigma, UK) and pH was adjusted to 7.35±0.05 (at 37°C). Glucose (10mM final concentration) (Fischer Scientific, USA), oligomycin (1µM final concentration (Sigma, UK) and 2-DG (100mM final concentration; Sigma), were added to individual ports to complete this assay.

### Metabolic and physiological monitoring

To assess metabolic gas exchange, mice were individually housed in indirect calorimetry cages (CLAMS cages, Columbus instruments). Mice previously maintained on a controlled light-dark light cycle were acclimatized to the cages for two 24-hour cycles, and oxygen consumption and carbon dioxide production was recorded at 10-minute intervals for at least a further three consecutive 24h light-dark cycles. Respiratory exchange ratio (RER) was derived from these measurements (VCO2/VO2), as was energy expenditure. For measurement of food intake genotype-matched co-housed mice were placed in a Sable System for a full week on a controlled 24-hour light-dark cycle. Following a two-day acclimatization period, food intake was measured for at least three consecutives 24h light-dark cycles.

### RT-PCR

Total RNA was purified using the RNeasy Micro Kit (Qiagen) and cDNA was prepared using the high capacity cDNA reverse transcription kit (Applied Biosystems). Real-time qPCR was performed with the real-time PCR StepOnePlus system (Applied Biosystems). *Nr1d1* (Mm00520708_m1), *Hk2* (Mm00443385_m1), *Hmgcs* (Mm01304569_m1) and *Xbp1* (Mm00457358_m1) were detected with commercial Taqman probe assays (Applied Biosystems). Remaining assays were performed either with TaqMan based chemistry (Applied Bio Systems) or with LightCycler 480 SYBR Green I Master Mix (Roche), using the primers and probes detailed in Supplementary Table 2.

### Bulk RNA sequencing

RNA was isolated from sort-purified cells, as above, and library preparation and bulk RNA sequencing was performed commercially with Novogene (UK) Company Ltd. Briefly, normalised RNA was used to generate libraries using NEB Next Ultra RNA library Prep Kit (Illumina). Indices were included to multiplex samples and mRNA was purified from total RNA using poly-T oligo-attached magnetic beads. After fragmentation, the first strand cDNA was synthesised using random hexamer primers followed by second strand cDNA synthesis. Following end repair, A-tailing, adaptor ligation and size section libraries were further amplified and purified and insert size validated on an Agilent 2100, and quantified using quantitative PCR (qPCR). Libraries were then sequenced on an Illumina NovaSeq 6000 S4 flowcell with PE150 according to results from library quality control and expected data volume. RNA Seq data are available via the GEO repository (Accession numbers, GSE175637, GSE175609).

### Bacterial 16S PCR

Bacteria from fecal pellets were extracted and normalised to fecal weight, and DNA was isolated using the DNeasy Powerlyser Powersoil Kit (Qiagen) according to manufacturer’s instructions. Bacterial DNA samples were amplified using the following primers: universal 16S forward 5’ GCA GGC CTA ACA CAT GCA AGT C 3’ and reverse 5’ CTG CTG CCT CCC GTA GGA GT 3’. Real time PCR was performed with the StepOnePlus Real-Time PCR system using SYBR Green Master Mix (Applied Biosystems). Bacterial copy number was calculated using a standard curve of known CFU and DNA concentrations from *S. aureus* cultures.

### 16S rRNA sequencing

Bacterial DNA from fecal bacteria was isolated using the PowerSoil DNA Isolation Kit (Qiagen, Netherlands) according to the manufacturer’s instructions. Pre-amplification of the V3V4 region of 16S rRNA was performed by PCR in triplicate using 2xKAPA HiFi Hot Start ReadyMix (Roche) using primer pairs containing adaptor sequences, as follows: 16S Amplicon PCR Forward Primer = 5'TCGT CGGCAGCGTCAGATGTGTATAAGAGACAGCCTACGGGNGGCWGCAG; 16S Amplicon PCR Reverse Primer = 5' GTCTCGTGGGCTCGGAGATGTGTATAAGAGACAGGACT ACHVGGGTATCTAATCC. Following this, AMPure XP beads (Fisher Scientific) were used to purify the 16S V3V4 amplicon away from free primers and primer dimer species, according to the manufacturer’s protocol. Illumina sequencing adapters were then attached using the Nextera XT Index Kit (Illumina Inc, USA), according to the manufacturer’s instructions. DNA libraries were then further purified using AMPure XP beads. DNA libraries were then quantified, normalised and pooled together for 16S sequencing via the Illumina MiSeq platform (Illumina, USA) at the University of Manchester. 16S rRNA Seq data are available via the ENA repository (Accession number, PRJEB53218). IgA-Seq was performed as described previously (38), and sequencing performed at the University of Liverpool.

### Shotgun Metagenomics

Shotgun metagenomics was performed commercially by CosmosID. Briefly, microbial DNA was extracted from fecal pellets and quantified using Qubit 4 fluorometer and HS Assay Kit (Thermofisher Scientific). DNA libraries were prepared using the Nextera XT DNA Library Preparation Kit and Nextera Index Kit (Illumina) following the manufacturer’s protocol with minor modifications. The standard protocol was used for a total DNA input of 1ng. Genomic DNA was fragmented using a proportional amount of Illumina Nextera XT fragmentation enzyme. Combinatory dual indexes were added to each sample followed by 12 cycles of PCR amplification. DNA libraries were then purified using AMPure magnetic beads (Beckman Coulter) and eluted in Qiagen EB buffer. DNA libraries were re-quantified and pooled together for sequencing via the Illimunia HiSeqX. Raw reads from metagenomics samples were analysed by CosmosID metagenomic software (CosmosID Inc., Rockville, MD, USA) to identify microbes to the strain level and a high-performance data mining k-mer algorithm was employed alongside highly curated dynamic comparator databases to rapidly disambiguate short reads into related genomes and genes.

### Functional profiling of shotgun metagenomic data

Following initial QC, adapter trimming and preprocessing of metagenomic sequencing reads were performed using BBduk. The quality-controlled reads were then subjected to a translated search using Diamond against a comprehensive and non-redundant protein sequence database, UniRef 90. The mapping of metagenomic reads to gene sequences were weighted by mapping quality, coverage and gene sequence length to estimate community wide weighted gene family abundances. Gene families are then annotated to MetaCyc reactions (Metabolic Enzymes) to reconstruct and quantify MetaCyc metabolic pathways in the community. Furthermore, the UniRef_90 gene families were regrouped to GO terms to generate an overview of community function. To facilitate comparisons across multiple samples with different sequencing depths, the abundance values were normalized using Total-sum scaling (TSS) normalization to produce "Copies per million" units.

### Bioinformatics

Where indicated bioinformatic analyses of data were performed via commercial platforms. For analysis of bulk RNA seq data differential gene expression analyses were performed in R (version 4.0.2) using RStudio Version 1.2.5033 (RStudio, Inc). Raw non-normalised counts were imported into R and subsequently analysed using the DESeq2 package (62), using the default pipeline. Genes with a total of fewer than ten counts across all samples were removed, and normalisation was calculated using the DESeq() function with default parameters for estimating size factors and dispersions. Differential expression was then calculated using the results() function with the default parameters. Genes with a significance value of less than 0.01 after correction for multiple comparisons using the Benjamini-Hochberg method were defined as “differentially expressed” and taken forward for further analysis. In some cases heatmaps were generated from normalised counts using the counts (normalised = TRUE) function followed by scaling and centring. Hierarchical clustering of genes was then computed using the ComplexHeatmap package (63). In other cases, clustering and normalised counts were then exported to excel and plotted in Graphpad Prism.

### Metabolomics

The metabolic profiles of fecal samples were measured using ^1^H nuclear magnetic resonance (NMR) spectroscopy as previously described (64). Briefly, fecal samples (30 mg) were defrosted and combined with 600µL of water and zirconium beads (0.45 g). Samples were homogenized with a Precellys 24 instrument (45 s per cycle, speed 6500, 2 cycles) and spun at 14,000 *g* for 10 minutes. The supernatants (400µL) were combined with 250µL phosphate buffer (pH 7.4, 100% D_2_O, 3 mM NaN_3_, and 1 mM of 3-(trimethyl-silyl)-[2,2,3,3-^2^H4]-propionic acid [TSP] for the chemical shift reference at δ0.0) before centrifugation at 14,000 *g* for 10 minutes, and then transferred to 5 mm NMR tubes for analysis on a Bruker 700 MHz spectrometer equipped with a cryoprobe (Bruker Biospin, Karlsruhe, Germany) operating at 300 K. ^1^H NMR spectra were acquired for each sample using a standard one-dimensional pulse sequence using the first increment of the NOE pulse sequence for water suppression as previously described (65). Raw spectra were phased, baseline corrected and calibrated to TSP using Topspin 3.2 (Bruker Biospin) and then digitized in a Matlab environment (Version 2018; Mathworks Inc, USA) using in-house scripts. Redundant spectral regions (related to water and TSP resonance) were removed and the spectral data was manually aligned and normalized to the probabilistic quotient using in-house Matlab scripts. The peak integrals (relating to relative abundance) for metabolites of interest were calculated for each sample.

### Statistical analyses

Statistical analysis of rhythmicity was calculated via JTK_Cycle analysis (66) of double plotted data sets using an established R pipeline. Data sets were tested for normality using a Shapiro-Wilks test to determine Gaussian distribution. For two-way comparisons statistics were performed by t-test or Mann-Whitney test, as otherwise indicated in the figure legend. For comparisons of three or more groups, or select circadian data sets, a Kruskal Wallis test, One-Way ANOVA or Two-Way ANOVA was performed as appropriate and as indicated in the figure legends. Values for statistical analyses are additionally reported in Supplementary Data File 4. Data is shown as standard error +/- mean.

## Supplementary Material

Supplementary Figures

## Data Availability

Data are included in the main text or supplementary materials and data files. Sequencing data sets are available via GEO (Accession numbers, GSE175637, GSE175609) and the ENA repository (Accession number, PRJEB53218). Mouse strains reported in this study are available via commercial vendors (Jackson laboratories) or by collaborative agreement with the respective co-author, as indicated in the methods.

## One-Sentence Summary

Diurnal rhythms in intestinal IgA act to cross-regulate oscillations in the abundance of commensal microbes to foster mutualism.
