# Supplementary material for: Rhythmicity of Intestinal IgA Responses Confers Oscillatory Commensal Microbiota Mutualism
Source: Sci Immunol. Author manuscript; Available in PMC 2022 Sep 29. (PMC7613662; doi:10.1126/sciimmunol.abk2541)
Supplement: Supplementary Figures [file EMS153855-supplement-Supplementary_Figures.docx]

**Fig. S1. Rhythms in secretory IgA are not associated with diurnal oscillatons in intestinal plasma cell numbers or Peyer’s patch responses.** A) IgA concentrations normalised to total protein concentrations of feces, serially sampled from C57BL/6 mice at five 6 hour intervals over a circadian day (ZT 0, 6, 12, 18, 0), *n*=10 (pooled from two independent experimental cohorts). Data representative of at least 2 independent experiments. B-D) Quantification of B) small intestinal lamina propria IgA^+^ PC cell numbers, C) colon lamina propria frequencies IgA^+^ PC (of live CD45^+^ cells) and D) associated cell numbers. B-D *n=*10 and representative of pooled data from two independent experiments. E) Representative flow plots indicating gating strategy for identification of Germinal Center (GC) B cells and IgA class-switched GC B cells in the Peyer’s Patches. Cells pre-gated as Live CD45^+^CD3-CD5-NK1.1-CD11b-MHCII^+^B220^+^. F) Frequencies of total GC B cells within Peyer’s Patch B cell compartment and G) IgA^+^ cells amongst GC B cells, from C57BL/6 mice sacrificed at four 6 hour intervals over a circadian day (ZT 0, 6, 12, 18), *n*=10 (pooled from two independent experimental cohorts). H) Secretion of IgA into culture media after overnight culture of equal numbers of sort-purified small intestinal IgA^+^ PC, Peyer’s Patch-derived IgA^+^ B cells or IgD^+^ B cells. P values determined by JTK Cycle. All data shown as +/- SEM unless otherwise indicated, * p< 0.05, ** p< 0.01, *** p< 0.001, **** p< 0.0001.

**Fig. S2. Diurnal rhythmicity in IgA^+^ Plasma Cell function is associated with oscillatory activity in metabolic pathways and sensing of survival signals.** A) Z-score heatmap of top 50 significantly oscillatory genes identified in small intestinal IgA^+^ PC sort-purified at ZT0, 6, 12 and 18. Average values of *n*=5 mice per timepoint. B) Enrichment of significantly oscillatory genes (highlighted in red) in the Mevalonate pathway. C) Relative expression (fpkm) of selected genes from B. D) Representative image indicating significantly oscillatory genes associated with cholersterol biosynthesis and metabolism and E) relative expression (fpkm) of selected genes from D. F) Relative expression (fpkm) of additional genes associated with pathways highlighed in Figure 1H. G) Relative expression (fpkm) of additional genes associated with pathways higlighed in Figure 1I. H+I) Intracellular Xbp1s and J-L) CD138 protein expression in IgA^+^ PCs measured at ZT0 and ZT12 (data represenative of *n*=10 mice per group, data in I and L pooled from two independent experiments). P values determined by JTK Cycle, with the exception of Panels I and L determined via a parametic, unpaired t-test. All data shown as +/- SEM unless otherwise indicated, * p< 0.05, ** p< 0.01, *** p< 0.001, **** p< 0.0001. Panel B+D images created with BioRender.com.

**Fig. S3. Circadian clock gene expression and validation of a conditional *Arntl*- knockout mouse model.** A) Relative expression (fpkm) of circadian clock genes as identified by bulk RNA seq in Figure 1. B) RT-PCR validaton of steady state circadian clock phase in whole liver tissue samples from mice culled at ZT0, 6, 12 and 18 (ZT0 double plotted). *n=*3-4 and representative of two independent experiments. C) RT-PCR validation of circadian clock genes in B220^+^ IgD^+^ B cells isolated from Peyer’s patches and small intestinal lamina propria of C57BL/6 mice culled at ZT0, 6, 12 and 18 (ZT0 double plotted). *n=*10 and representative of pooled data taken from two independent experiments. D) *Arntl* and E) *Nr1d1* expression in small intestinal IgA^+^ PC sort-purified from *Mb1*^Cre/+^ x *Arntl*^fl/fl^ mice in comparison to *Mb1*^+/+^ x *Arntl*^fl/fl^ littermate control animals, *n*=3-4 mice per group and representative of two independent experiments. P values determined by JTK Cycle, with the exception of Panels D and E determined via a parametic, unpaired t-test. All data shown as +/- SEM unless otherwise indicated, * p< 0.05, ** p< 0.01, *** p< 0.001, **** p< 0.0001.

**Fig. S4. High fat diet disrupts diurnal IgA responses.** A) IgA concentrations normalised to total protein concentrations of feces, serially sampled from light-fed and dark-fed C57BL/6 mice at four 6 hour intervals over a circadian day (ZT 0, 6, 12, 18; ZT0 double plotted), *n*=9-10 (pooled from two independent experimental cohorts). Data representative of at least three independent experiments. B) Validation of circadian clock dysregulation by reverse feeding in whole liver tissue. RT-PCR analysis at ZT0 and ZT12 in samples taken from light-fed or dark-fed mice, *n*=5 per group, data representative of two independent experiments. Quantification of C) small intestinal IgA^+^ PC frequencies (% of total CD45^+^) or D) Peyer’s Patch IgA^+^ GC B cells (% of GC B cell compartment) in light-fed or dark-fed mice, *n*=10 per group, data pooled from two independent experiments. E) Body weight of mice, F) post-prandial blood glucose (*ad lib* fed, sampled at ZT1) and G) fasting glucose (following 8 hour fast) in normal chow and high fat diet fed mice. H) Food intake (grams per 3 minute interval) over a 24 hour day and I) percent of food intake in light period of mice fed normal chow or high fat diet fed and housed in metabolic cage, *n=6* per group. Representative of at least two independent experiments. P values determined by JTK Cycle (A), Two-Way ANOVA (B, E-G), and a parametric, unpaired t-test (I). All data shown as +/- SEM unless otherwise indicated, * p< 0.05, ** p< 0.01, *** p< 0.001, **** p< 0.0001.

**Fig. S5. IgA^+^ plasma cells exhibit elevated metabolic activity and comparable capacity to utilize metabolic substrates over the course of a circadian day.** A-J) Assays to determine metabolic activity of IgA^+^ PC, IgA^+^ B cells and IgD^+^ B cells from small intestinal lamina propria and Peyer’s patches. Data pooled from at least two independent experiments. A+B) Uptake of 2-NBDG, C+D) CD98 expression, E+F) Kynurenine uptake and G) Kynurenine uptake in the presence of the amino acid inhibitor BCH, or exogenous cold Leucine, H+I) LipidTox staining. J) Glycolytic stress test of sort-purified small intestinal-derived IgA+ PC and Peyer’s patch-derived IgD^+^ B cells by extracellular flux analysis. Data representative of two independent experiments. Comparison of K) 2-NBDG uptake, L) CD98 expression, M) Kynurenine uptake and N) glycolytic capacity in IgA^+^ PC at ZT 6 and ZT18. P values determined via a non-parametic Kruskal Wallis test. All data shown as +/- SEM unless otherwise indicated, statistical test performed as indicated in supplementary raw data file; * p< 0.05, ** p< 0.01, *** p< 0.001, **** p< 0.0001.

**Fig. S6. IgMi mouse validation and circadian rhythms in microbial abundance.** A+B) IgA+ PC frequencies in the small intestinal lamina propria (representative of *n*=10 mice, pooled from two independent experiments), and C) Fecal IgA measured at four circadian time points in SPF and Germ Free (GF) animals (*n*=5 mice per group, representative of two independent experiments). D) Fecal IgA in IgMi versus control littermate animals. E) IgA binding of fecal commensal baceria in IgMi and control littermates assessed by flow cytometry. F) Total bacterial abundance in WT and IgMi mice as assessed over four circadian time points (ZT0 double plotted). G+H) Relative abundance of *Akkermansia* spp. in IgMi and Ctrl mice assessed via G) global analysis or H) ZT analysis. I) Selected commensal bacteria exhibiting no significant oscillatory behaviour in IgMi or Ctrl animals. J) Selected commensal bacteria exhibiting significant oscillatory behaviour in Ctrl but not IgMi animals but not detected in IgA Seq analysis, related to Figure 4G and 4J. K) Selected commensal bacteria exhibiting a retained but significant phase shift in oscillatory behaviour between Ctrl and IgMi animals, related to Figure 4H, and L) Selected commensal bacteria exhibiting a significant oscillatory behaviour that is retained in both Ctrl and IgMi animals, related to Figure 4H. M+N) IgA binding to fecal bacteria as assessed by flow cytometry at four circadian time points (*n*=7 mice pooled from two independent experiments). P values determined via JTK Cycle with the exception of panels D, E and G determined via a non-parametric, unpaired Mann-Whitney test. All data shown as +/- SEM unless otherwise indicated, * p< 0.05, ** p< 0.01, *** p< 0.001, **** p< 0.0001.

**Fig. S7. Alterations in predicted microbial gene pathways in the absence of mucosal antibody.** A) Schematic of GO Term pathways related to Figure 5C and glucose metabolism. Steps highlighted in red indicate significant oscillatory steps and/or enzymatic activity. B) Selected individual data for pathways related to A. C) Selected individual data of microbial GO Term pathways either lost or gained in IgMi mice in comparison to Control animals, *n*=5 per group per timepoint and representative of a single experiment. P values determined by JTK analysis. All data shown as +/- SEM unless otherwise indicated, * p< 0.05, ** p< 0.01, *** p< 0.001, **** p< 0.0001. Panel A image created with BioRender.com.

**Fig. S8. Dysregulation of circadian metabolites in the absence of mucosal antibody.** A) Additional fecal-associated metabolites over four time points in IgMi and Ctrl animals. Related to Figure 5D. B) Measurement of food intake at 1 hour intervals over three consecutive days in IgMi and Ctrl animals, *n*=4 per group and representative of a single experiment. C) Measurement of Respiratory Exchange Ratio and D) Metabolic Rate, measured in CLAMS cages over three consecutive days in IgMi and Ctrl animals, *n*=2-4 per group and representative of two independent experiments. P values determined by JTK analysis. All data shown as +/- SEM unless otherwise indicated, * p< 0.05, ** p< 0.01, *** p< 0.001, **** p< 0.0001.

**Fig. S9. Schematic summary of circadian IgA regulation of rhythmic commensal microbiota mutualism.** Here we demonstrate diurnal oscllations in the secretion of IgA by small intestinal plasma cells. Oscillations in IgA requried the presence of the microbiota and were in part regulated by cell-intrinsic circadian clock machinery, but most markedly by feeding-associated cues. Oscillations in IgA act in part to regulate concurrent oscillations in the composition and relative abundance of commensal bacteria within the intestinal-resident microbiota and to influence the activity and function of gut microbes. These data suggest that there may be a complex and reciprocal relationship between the regulation of diurnal oscillations in both IgA and microbiota that are influenced by the diet and together act to coordinate mutualism between commensal microbes and the host. Image created with BioRender.com.

**Supplementary Table 1: List of Flow Cytometry Antibodies**

| **Target** | **Clone** | **Supplier** | **Cat #** | **RRID** |
| --- | --- | --- | --- | --- |
| anti-mouse CD3e | 506 145-2C11 | ThermoFisher | 45-0031 | AB_1107000 |
| anti-mouse CD5 | 53-7.3 | ThermoFisher | 45-0051 | AB_914334 |
| anti-mouse B220 | Ra3-6B2 | ThermoFisher | 47-0452 | AB_1518810 |
| anti-mouse CD11b | M1/70 | ThermoFisher | 63-0112 | AB_2637408 |
| anti-mouse H2-Ab1 | M5/114.15.2 | ThermoFisher | 48-5321 | AB_1272204 |
| anti-mouse CD45 | 30-F11 | BioLegend | 103151 | AB_2565884 |
| anti-mouse Fas | 15A7 | ThermoFisher | 53-0951 | AB_10671269 |
| anti-mouse GL7 | GL7 | BioLegend | 144606 | AB_2562185 |
| anti-mouse CD38 | 90 | ThermoFisher | 56-0381 | AB_657740 |
| anti-mouse CD98 | 4F2 | BioLegend | 128210 | AB_2254922 |
| anti-mouse CD19 | 1D3 | BD Biosciences | 563557 | AB_2722495 |
| anti-mouse IgA | mA-6E1 | ThermoFisher | 12-4204 | AB_465918 |
| anti-mouse IgD | 11-26c.2a | BioLegend | 405742 | AB_2571985 |
| anti-mouse Xbp1s | Q3-695 | BD Biosciences | 562821 | AB_2737817 |
| anti-mouse CD138 | 281-2 | BioLegend | 142513 | AB_2562197 |

**Supplementary Table 2: List of RT-PCR primers**

| **Target / chemistry** | **Primer Sequence** | |
| --- | --- | --- |
| *Gapdh*  (Taqman) | **Fwd:** | 5’ CAA TGT GTC CGT CGT CGA TCT 3’ |
|  | **Rev:** | 5’ GTC CTC AGT GTA GCC CAA GAT G 3’ |
|  | **Probe:** | 5’ CGT GCC GCC TGG AGA AAC CTG CC 3’ |
| *Arntl*  (Taqman) | **Fwd:** | 5’ CCA AGA AAG TAT GGA CAC AGA CAA A 3’ |
|  | **Rev:** | 5’ GCA TTC TTG ATC CTT CCT TGG T 3’ |
|  | **Probe:** | 5’ TGA CCC TCA TGG AAG GTT AGA ATA TGC AGA A 3’ |
| *Per2*  (Taqman) | **Fwd:** | 5’ GCC TTC AGA CTC ATG ATG ACA GA 3’ |
|  | **Rev:** | 5’ TTT GTG TGC GTC AGC TTT GG G 3’ |
|  | **Probe:** | 5’ ACT GCT CAC TAC TGC AGC CGC TCG T 3’ |
| *pIgR*  (SYBR) | **Fwd:** | 5’ CTG GGG AAG AGG GAT CCA GA 3’ |
|  | **Rev:** | 5’ ACT CCC TTC ACA ACA GAG CG 3’ |
| *bactin*  (SYBR) | **Fwd:** | 5’ TCCTATGTGGGTGACGAG 3’ |
|  | **Rev:** | 5’ CTCATTGTAGAAGGTGTGGTG 3’ |
